# Current Awareness on Comparative and Functional Genomics

**DOI:** 10.1002/cfg.58

**Published:** 2001-08


**Comparative and Functional Genomics**
*Comp. Funct. Genom.* 2001; **2:** 265–272
**DOI**:10.1002/cfg.58

### Current awareness on comparative and functional genomics

### 1 Reviews & symposia

2001. Special database issue. *Nucleic Acids Res* **29:** (1).

2000. Special issue on the genomic exploration of yeasts. *FEBS Lett* **487:** (1).

2000. Special issue on the post-genome era (Articles in French). *Biofutur* (206).

2000. Special issue: Human Genomics: The Basis of the Medicine of Tomorrow - Validating and using Pharmacogenomics - Second IFCC-Roche Diagnostics Conference, Kyoto, Japan, 16-19 April 2000. *Clin Chem Lab Med* **38:** (9).

2000. Special issue: Pharmacogenomics. *Eur J Pharmacol* **410:** (2-3).

Bell AC, West AG, Felsenfeld G*. 2001. *NIDDKD, Bethesda, Md 20892, USA. Gene regulation: - Insulators and boundaries: Versatile regulatory elements in the eukaryotic genome (Review). *Science* **291:** (5503) 447.

Bernardi G. 2000. Stn Zool A Dohrn, Lab Evoluzione Mol, IT-80121 Naples, Italy. The compositional evolution of vertebrate genomes. *Gene* **259:** (1-2) 31.

Brady G. 2000. Univ Manchester, Sch Biol Sci, G.38 Stopford Bldg, Oxford Rd, Manchester M13 9PT, England. Expression profiling of single mammalian cells: Small is beautiful. *Yeast* **17:** (3) 211.

Broder C, Venter JC. 2000. Celera Genomics Corp, 45 West Gude Dr, Rockville, Md 20850, USA. Whole genomes: The foundation of new biology and medicine - Commentary. *Curr Opin Biotechnol* **11:** (6) 581.

Brussow H, Desiere F. 2000. Nestec Ltd, Nestle Res Ctr, Vers Chez Les Blanc, CH-1000 Lausanne 26, Switzerland. Comparative phage genomics and the evolution of Siphoviridae: Insights from dairy phages (Microreview). *Mol Microbiol* **39:** (2) 213.

Bumol TF, Watanabe AM. 2001. Lilly Corp Ctr, Res Labs, Res Technol & Prot, Indianapolis, In 46285, USA. Genetic information, genomic technologies, and the future of drug discovery. *JAMA* **285:** (5) 551.

Celniker SE. 2000. Lawrence Berkeley Natl Lab, Drosophila Genome Project, Berkeley, Ca 94720, USA. The *Drosophila* genome. *Curr Opin Genet Dev* **10:** (6) 612.

Cockett M, Dracopoli N, Sigal E*. 2000. *Bristol Myers Squibb Co, Applied Genomics, 311 Pennington-Rocky Hill Rd, Pennington, NJ 08534, USA. Applied genomics: Integration of the technology within pharmaceutical research and development. *Curr Opin Biotechnol* **11:** (6) 602.

Cogoni C, Macino G. 2000. Univ Roma La Sapienza, Sezione Genet Mol, IT-00161 Rome, Italy. Post-transcriptional gene silencing across kingdoms. *Curr Opin Genet Dev* **10:** (6) 638.

Degtyarenko K. 2000. Wellcome Trust Genome, European Bioinformat Inst, EMBL Outstn, Cambridge CB10 1SD, England. Bioinorganic motifs: Towards functional classification of metalloproteins (Review). *Bioinformatics* **16:** (10) 851.

Dougherty ER. 2001. Texas A&M Univ, Dept Elect Engn, College Station, Tx 77843, USA. Small sample issues for microarray-based classification (Review). *Comp Funct Genom* **2:** (1) 28.

Dovichi NJ, Zhang JZ. 2000. Univ Alberta, Dept Chem, Edmonton, Alberta, Canada T6G 2G2. How capillary electrophoresis sequenced the human genome. *Angew Chem Int Ed* **39:** (24) 4463.

Dow JM, Daniels MJ*. 2000. *John Innes Ctr, Sainsbury Lab, Norwich Res Park, Colney, Norwich NR4 7UH, England. *Xylella* genomics and bacterial pathogenicity to plants (Review). *Yeast* **17:** (4) 263.

Dunham I. 2000. Wellcome Trust Genome Campus, The Sanger Ctr, Hinxton, Cambridge CB10 1SA, England. The gene guessing game (Review). *Yeast* **17:** (3) 218.

Eisen JA. 2000. Inst Genomic Res, 9712 Med Ctr Dr, Rockville, Md 20850, USA. Horizontal gene transfer among microbial genomes: New insights from complete genome analysis. *Curr Opin Genet Dev* **10:** (6) 606.

Ekker SC. 2000. Univ Minnesota, Dept Genet, Cell Biol & Dev, Beckman Ctr Transposon Res, 6-160 Jackson Hall, 321 Church St SE, Minneapolis, Mn 55455, USA. Morphants: A new systematic vertebrate functional genomics approach (Review). *Yeast* **17:** (4) 302.

Erlandsen H, Abola EE, Stevens RC. 2000. Scripps Clin & Research Inst, Department Mol Biol, La Jolla, Ca 92037, USA. Combining structural genomics and enzymology: Completing the picture in metabolic pathways and enzyme active sites. *Curr Opin Struct Biol* **10:** (6) 719.

Ferrier DEK, Holland PWH. 2001. Univ Reading, Sch Anim & Microbial Sci, Whiteknights, Reading RG6 6AJ, England. Ancient origin of the Hox gene cluster. *Nat Rev Genet* **2:** (1) 33.

Galliot B. 2000. Univ Geneva, Dept Zool & Anim Biol, 30 Quai Ernest Ansermet, CH-1211 Geneva 4, Switzerland. Conserved and divergent genes in apex and axis development of cnidarians. *Curr Opin Genet Dev* **10:** (6) 629.

Gautier C. 2000. Univ Lyon 1, Lab Biometry & Evolutionary Biol, 43 Blvd 11 Nov, FR-69622 Villeurbanne, France. Compositional bias in DNA. *Curr Opin Genet Dev* **10:** (6) 656.

Graham A. 2000. KCL, Mol Neurobiol Grp, 4th Floor New Hunts Hse, Guys Campus, London SE1 9RT, England. The evolution of the vertebrates: Genes and development. *Curr Opin Genet Dev* **10:** (6) 624.

Gurtler V, Mayall BC. 2001. Austin & Repatriat Med Ctr, Dept Microbiol, Austin Campus, Studley Rd, Heidelberg, Vic 3084, Australia. Genomic approaches to typing, taxonomy and evolution of bacterial isolates (Review). *Int J Syst Evol Microbiol* **51:** (1) 3.

Hayakawa T, Rohrmann GF*, Hashimoto Y. 2000. *Oregon State Univ, Dept Microbiol, Nash Hall 220, Corvallis, Or 97331, USA. Patterns of genome organization and content in lepidopteran baculoviruses (Minireview). *Virology* **278:** (1) 1.

Horrocks P, Bowman S, Kyes S, Waters AP, Craig A*. 2000. *Univ Liverpool, Sch Trop Med, Pembroke Pl, Liverpool L3 5QA, England. Entering the post-genomic era of malaria research. *Bull World Health Organ* **78:** (12) 1424.

Johnston M. 2000. Washington Univ, Dept Genet, 660 Euclid Ave, St Louis, Mo 63113, USA. The yeast genome: On the road to the Golden Age. *Curr Opin Genet Dev* **10:** (6) 617.

Justice MJ. 2000. Baylor Coll Med, Dept Biol & Human Genet, 1 Baylor Plaza, Houston, Tx 77030, USA. Capitalizing on large-scale mouse mutagenesis screens (Review). *Nat Rev Genet* **1:** (2) 109.

Kaminski N. 2000. Chaim Sheba Med Ctr, Inst Resp Med, Funct Genomics Unit, IL-52621 Tel Hashomer, Israel. Bioinformatics: A user's perspective (Minireview). *Am J Respir Cell Mol Biol* **23:** (6) 705.

Knight RD, Freeland SJ, Landweber LF*. 2001. *Princeton Univ, Dept Ecol & Evolut Biol, Princeton, NJ 08544, USA. Rewiring the keyboard evolvability of the genetic code. *Nat Rev Genet* **2:** (1) 49.

Kusnirikova P, Cellarova E. 2000. Safarik Univ, Fac Sci, Dept Expt Bot & Genet, Manesova 23, SK-04167 Kosice, Slovakia. Sequence databases for molecular biologists (Review). *Biologia* **55:** (6) 617.

Le TH, Blair D, McManus DP*. 2000. *Queensland Institute Med Research, Mol Parasitol Unit, 300 Herston Road, Brisbane, Qld 4029, Australia. Mitochondrial genomes of human helminths and their use as markers in population genetics and phylogeny (Review). *Acta Trop* **77:** (3) 243.

Li JY, Boado RJ, Pardridge WM*. 2001. *UCLA, Dept Med, Los Ange-

In order to keep subscribers up-to-date with the latest developments in their field, this current awareness service is provided by John Wiley & Sons and contains newly-published material on comparative and functional genomics. Each bibliography is divided into 16 sections. 1 Reviews & symposia; 2 General; 3 Large-scale sequencing and mapping; 4 Evolutionary genomics; 5 Comparative genomics; 6 Pathways, gene families and regulons; 7 Pharmacogenomics; 8 Large-scale mutagenesis programmes; 9 Functional genomics; 10 Transcriptomics; 11 Proteomics; 12 Protein structural genomics; 13 Metabolomics; 14 Genomic approaches to development; 15 Technological advances; 16 Bioinformatics. Within each section, articles are listed in alphabetical order with respect to author. If, in the preceding period, no publications are located relevant to any one of these headings, that section will be omitted.

Copyright © 2001 John Wiley & Sons, Ltd.

les, Ca 90095, USA. Blood-brain barrier genomics. *J Cereb Blood Flow Metab* **21:** (1) 61.

Li M. 2000. Johns Hopkins Univ, Dept Physiol, 725 Nth Wolfe St, Baltimore, Md 21205, USA. Applications of display technology in protein analysis (Review). *Nat Biotechnol* **18:** (12) 1251.

Lockhart DJ, Barlow C. 2001. Salk Inst Biol Studies, Genet Lab, 10010 Nth Torrey Pines Rd, La Jolla, Ca 92037, USA. Expressing what's on your mind: DNA arrays and the brain. *Nat Rev Neurosci* **2:** (1) 63.

Loferer H. 2000. Biotech AG, Fraunhoferstr 20, DE-82152 Martinsried, Germany. Mining bacterial genomes for antimicrobial targets (Review). *Mol Med Today* **6:** (12) 470.

Luzi L, Confalonieri S, Di Fiore PP, Pelicci PG. 2000. Ist Europeo Oncol, Dept Expt Oncol, via Ripamonti 435, IT-20141 Milan, Italy. Evolution of Shc functions from nematode to human. *Curr Opin Genet Dev* **10:** (6) 668.

Mahan MJ, Heithoff DM, Sinsheimer RL, Low DA. 2000. Univ Calif, Dept Mol Cellular & Dev Biol, Santa Barbara, Ca 93106, USA. Assessment of bacterial pathogenesis by analysis of gene expression in the host. *Annu Rev Genet* **34:** 139.

Makalowski W. 2000. NIH, Natl Lib Med, Natl Ctr Biotechnol Informat, Bethesda, Md 20894, USA. Genomic scrap yard: How genomes utilize all that junk. *Gene* **259:** (1-2) 61.

Mir KU, Southern EM. 2000. Univ Oxford, Dept Biochem, Oxford OX1 3QU, England. Sequence variation genes and genomic DNA: Methods for large-scale analysis. *Annu Rev Genomic Hum Genet* **1:** 329.

Newburger PE, Subrahmanyam YVBK, Weissman SM. 2000. Univ Massachusetts, Dept Pediat, 373 Plantation St, Worcester, Ma 01605, USA. Global analysis of neutrophil gene expression. *Curr Opin Hematol* **7:** (1) 16.

Nock S, Wagner P. 2000. Zyomyx Inc, 3911 Trust Way, Hayward, Ca 94545, USA. Proteomics: The post-genome revolution (German). *Chem Unserer Zeit* **34:** (6) 348.

Ohta T. 2000. Natl Inst Genet, Shizuoka 411 8540, Japan. Evolution of gene families. *Gene* **259:** (1-2) 45.

Pannuti A, Lucchesi JC. 2000. Emory Univ, Dept Biol, 1510 Clifton Rd, Atlanta, Ga 30322, USA. Recycling to remodel: Evolution of dosage-compensation complexes. *Curr Opin Genet Dev* **10:** (6) 644.

Paw BH, Zon LI*. 2000. *Children's Hosp, Div Hematol Oncol, 320 Longwood Ave, Boston, Ma 02115, USA. Zebrafish: A genetic approach in studying hematopoiesis. *Curr Opin Hematol* **7:** (2) 79.

Pennacchio LA, Rubin EM*. 2001. *Lawrence Berkeley Lab, Genome Sci Dept, 1 Cyclotron Rd, Berkeley, Ca 94720, USA. Genomic strategies to identify mammalian regulatory sequences. *Nat Rev Genet* **2:** (2) 100.

Philippe H, Germot A, Moreira D. 2000. Equipe Phylogenie Bioinformat & Genome, UMR CNRS 7622, 9 Quai St Bernard, Case 24, FR-75252 Paris 05, France. The new phylogeny of eukaryotes. *Curr Opin Genet Dev* **10:** (6) 596.

Pollock DD, Eisen JA, Doggett NA, Cummings MP. 2000. Louisiana State Univ, Dept Biol Sci, Baton Rouge, La 70803, USA. A case for evolutionary genomics and the comprehensive examination of sequence biodiversity. *Mol Biol Evol* **17:** (12) 1776.

Prescott DM. 2000. Univ Colorado, Dept Mol Cellular & Dev Biol, Boulder, Co 80309, USA. Genome gymnastics: Unique modes of DNA evolution and processing in ciliates. *Nat Rev Genet* **1:** (3) 191.

Rininger JA, Di Pippo VA, Gould-Rothberg BE. 2000. Curagen Corp, 555 Long Wharf Dr, New Haven, Ct 06511, USA. Differential gene expression technologies for identifying surrogate markers of drug efficacy and toxicity. *Drug Discov Today* **5:** (12) 560.

Sanderfoot AA, Assaad FF, Raikhel NV*. 2000. *Michigan State Univ, Plant Res Lab, East Lansing, Mi 48824, USA. The *Arabidopsis* genome. An abundance of soluble *N*-ethylmaleimide-sensitive factor adaptor protein receptors. *Plant Physiol* **124:** (4) 1558.

Schulze A, Downward J. 2000. ICRF Signal Transduct Lab, 44 Lincolns Inn Fields, London WC2A 3PX, England. Analysis of gene expression by microarrays: Cell biologist's gold mine or minefield? *J Cell Sci* **113:** (23) 4151.

Schwarz DG. 2000. Univ Nth Carolina, Dept Genet, CB 7264, 102 Mason Farm Rd, Chapel Hill, NC 27599, USA. DNA chips and development: Prospects and problems. *Genesis* **28:** (3-4) 134.

Searls DB. 2000. SmithKline Beecham Pharmaceut, Bioinformat Dept, King of Prussia, Pa 19406, USA. Bioinformatics tools for whole genomes. *Annu Rev Genomic Hum Genet* **1:** 251.

Shiffman D, Porter JG. 2000. CV Therapeut Inc, 3172 Porter Dr, Palo Alto, Ca 94304, USA. Gene expression profiling of cardiovascular disease models. *Curr Opin Biotechnol* **11:** (6) 598.

Sicheritz-Ponten T, Andersson SGE*. 2001. *Uppsala Univ, Dept Mol Evolut, Ctr Evolut Biol, SE-75236 Uppsala, Sweden. A phylogenomic approach to microbial evolution. *Nucleic Acids Res* **29:** (2) 545.

Snijders AM, Meijer GA, Brakenhoff RH, Van den Brule AJC, Van Diest PJ*. 2000. *Vrije Univ Hosp, Dept Pathol, Box 7057, NL-7057 MB Amsterdam, The Netherlands. Microarray techniques in pathology: Tool or toy? (Review). *J Clin Pathol-Mol Pathol* **53:** (6) 289.

Sorrentino V, Barone V, Rossi D. 2000. Ist Sci San Raffaele, DIBIT, via Olgettina 58, IT-20132 Milan, Italy. Intracellular $Ca^{2+}$ release channels in evolution. *Curr Opin Genet Dev* **10:** (6) 662.

Stainier DYR. 2001. Univ Calif, Dept Biochem & Biophys, 513 Parnassus Ave, San Francisco, Ca 94143, USA. Zebrafish genetics and vertebrate heart formation. *Nat Rev Genet* **2:** (1) 39.

Steffen DL, Levine AE, Yarus S, Baasiri RA, Wheeler DA. 2000. Biomedical Computing Inc, Houston, Tx 77005, USA. Digital reviews in molecular biology: Approaches to structured digital publication. *Bioinformatics* **16:** (7) 639.

Stockwell BR. 2000. Whitehead Inst Biomed Res, 9 Cambridge Ctr, Cambridge, Ma 02142, USA. Chemical genetics: Ligand-based discovery of gene function (Review). *Nat Rev Genet* **1:** (2) 116.

Sullivan DC, Hoffman JM. 2001. NCI, Room 6070, 6130 Executive Blvd, Bethesda, Md 20892, USA. *In vivo* imaging of gene expression. *Semin Radiat Oncol* **11:** (1) 37.

Thorne JL. 2000. NC State Univ, Program Stat Genet, Box 8203, Raleigh, NC 27695, USA. Models of protein sequence evolution and their applications. *Curr Opin Genet Dev* **10:** (6) 602.

Thornton JW, De Salle R. 2000. Columbia Univ, Dept Biol Sci, New York, NY 10027, USA. Gene family evolution and homology: Genomics meets phylogenetics. *Annu Rev Genomic Hum Genet* **1:** 41.

Torres MJ, Matvienko M, Yoder JI. 2000. Univ Calif, Dept Veg Crops, 1 Shields Ave, Davis, Ca 95616, USA. Agricultural genomics and subterranean plant-plant communications. *J Cell Biochem* **80:** (2) 203.

Watson SJ, Meng F, Thompson RC, Akil H. 2000. Univ Michigan, Mental Hlth Res Inst, 205 Zina Pitcher Pl, Ann Arbor, Mi 48109, USA. The "chip" as a specific genetic tool (Review). *Biol Psychiatry* **48:** (12) 1147.

Wren BW. 2000. London Sch Hyg & Trop Med, Dept Infect & Trop Dis, Keppel St, London WC1E 7HT, England. Microbial genome analysis: Insights into virulence, host adaptation and evolution. *Nat Rev Genet* **1:** (1) 30.

Zivy M, De Vienne D. 2000. Univ Paris Sud, INA PG/INRA, Stn Genet Vegetale, FR-91190 Gif-sur-Yvette, France. Proteomics: A link between genomics, genetics and physiology. *Plant Mol Biol* **44:** (5) 575.

### 3 Large-scale sequencing and mapping

Abrahamsen MS. 2001. Univ Minnesota, Vet PathoBiol, 1988 Fitch Ave, St Paul, Mn 55108, USA. *Cryptosporidium parvum* genome project (Conference Paper). *Comp Funct Genom* **2:** (1) 19.

Afonso CL, Tulman ER, Lu Z, Zsak L, Rock DL, Kutish GF*. 2001. *USDA/ARS, Plum Isl Anim Dis Ctr, POB 848, Greenport, NY 11944, USA. The genome of turkey herpesvirus. *J Virol* **75:** (2) 971.

Aguero F, Verdun RE, Frasch ACC, Sanchez DO*. 2000. *Univ Nacl Gen San Martin, CONICET, Inst Invest Biotecnol, AR-1650 San Martin, Buenos Aires, Argentina. A random sequencing approach for the analysis of the *Trypanosoma cruzi* genome: General structure, large gene and repetitive DNA families, and gene discovery. *Genome Res* **10:** (12) 1996.

Anderson JB, Wickens C, Khan M, Cowen LE, Federspiel N, Jones T, Kohn LM. 2001. Univ Toronto, Dept Bot, 3359 Mississauga Rd, Mississauga, Ontario, Canada L5L 1C6. Infrequent genetic exchange and recombination in the mitochondrial genome of *Candida albicans*. *J Bacteriol* **183:** (3) 865.

Biagini P, Gallian P, Attoui H, Touinssi M, Cantaloube JF, De Micco P, De Lamballerie X*. 2001. *Fac Med Marseille, Lab Virol Mol Trop & Transfus, EA 871, Unite Virus Emergents, 27 Blvd Jean Moulin, FR-13005 Marseille, France. Genetic analysis of full-length genomes and subgenomic sequences of TT virus-like mini virus human isolates. *J Gen Virol* **82:** (2) 379.

Chibana H, Beckerman JL, Magee PT. 2000. Univ Minnesota, Dept Genet Cell Biol & Dev, St Paul, Mn 55108, USA. Fine-resolution

Copyright © 2001 John Wiley & Sons, Ltd. 

physical mapping of genomic diversity in *Candida albicans*. *Genome Res* **10:** (12) 1865.

Drenkard E, Richter BG, Rozen S, Stutius LM, Angell NA, Mindrinos M, Cho RJ, Oefner PJ, Davis RW, Ausubel FM*. 2000. *Harvard Univ, Dept Genet, Boston, Ma 02114, USA. A simple procedure for the analysis of single nucleotide polymorphisms facilitates map-based cloning in *Arabidopsis*. *Plant Physiol* **124:** (4) 1483.

Hill F, Gemund C, Benes V, Ansorge W, Gibson TJ. 2000. Avidis SA, FR-63730 St Beauzire, France. An estimate of large-scale sequencing accuracy. *EMBO Rep* **1:** (1) 29.

Perna NT, Plunkett G, Burland V, Mau B, Glasner JD, Rose DJ, Mayhew GF, Evans PS, Gregor J, Kirkpatrick HA *et al*. 2001. Univ Wisconsin, Genome Ctr, Madison, Wi 53706, USA. Genome sequence of enterohaemorrhagic *Escherichia coli* O157:H7. *Nature* **409:** (6819) 529.

Prescott DM, Dizick SJ. 2000. Univ Colorado, Dept Mol Cellular & Dev Biol, Boulder, Co 80309, USA. A unique pattern of intrastrand anomalies in base composition of the DNA in hypotrichs. *Nucleic Acids Res* **28:** (23) 4679.

Sanchez DO, Zandomeni RO, Cravero S, Verdun RE, Pierrou E, Faccio P, Diaz G, Lanzavecchia S, Aguero F, Frasch ACC, Andersson SG, Rossetti OL, Grau O, Ulgalde RA* . 2001. *Univ Nacl Gen San Martin, Inst Invest Biotecnol, Inst Tecnol Chascomus, AR-1650 San Martin, Buenos Aires, Argentina. Gene discovery through genomic sequencing of *Brucella abortus. Infect Immun* **69:** (2) 865.

Yu A, Zhao CF, Fan Y, Jang WH, Mungall AJ, Deloukas P, Olsen A, Doggett NA, Ghebranious N, Broman KW, Weber JL*. 2001. *Marshfield Med Res Fdn, Ctr Med Genet, Marshfield, Wi 54449, USA. Comparison of human genetic and sequence-based physical maps. *Nature* **409:** (6822) 951.

### 4 Evolutionary genomics

Alba MM, Das RJ, Orengo CA, Kellam P*. 2001. *UCL, Dept Immunol & Mol Pathol, Wohl Virion Ctr, London W1T 4JF, England. Genomewide function conservation and phylogeny in the Herpesviridae. *Genome Res* **11:** (1) 43.

Cao Y, Fujiwara M, Nikaido M, Okada N, Hasegawa M*. 2000. *Inst Stat Math, 4-6-7 Minami-Azaabu, Minato ku, Tokyo 106 8569, Japan. Interordinal relationships and timescale of eutherian evolution as inferred from mitochondrial genome data. *Gene* **259:** (1-2) 149.

Cooper A, Lalueza-Fox C, Anderson S, Rambaut A, Austin J, Ward R. 2001. Univ Oxford, Dept Biol Anthropol, Oxford OX1 6UE, England. Complete mitochondrial genome sequences of two extinct moas clarify ratite evolution. *Nature* **409:** (6821) 704.

De Villena FPM, De la Casa-Esperon E, Sapienza C. 2000. Temple Univ, Fels Inst Canc Res & Mol Biol, 3307 Nth Broad St, Philadelphia, Pa 19140, USA. Natural selection and the function of genome imprinting: Beyond the silenced minority. *Trends Genet* **16:** (12) 573.

Funk DJ, Helbling L, Wernegreen JJ, Moran NA. 2000. Vanderbilt Univ, Dept Biol, Nashville, Tn 37235, USA. Intraspecific phylogenetic congruence among multiple symbiont genomes. *Proc Royal Soc Lond Ser B* **267:** (1461) 2517.

Groth C, Petersen RF, Piskur J*. 2000. *Tech Univ Denmark, Dept Microbiol, Bldg 301, DK-2800 Lyngby, Denmark. Diversity in organization and the origin of gene orders in the mitochondrial DNA molecules of the genus *Saccharomyces*. *Mol Biol Evol* **17:** (12) 1833.

Kawashima T, Amano N, Koike H, Makino S, Higuchi S, Kawashima-Ohya Y, Watanabe K, Yamazaki M, Kanehori K, Kawamoto T, Nunoshiba T, Yamamoto Y, Aramaki H, Makino K, Suzuki M*. 2000. *Natl Inst Biosci & Human Technol, Core Res Evolut Sci & Technol Ctr Struct Biol, 1-1 Higashi, Tsukuba, Ibaraki 305 0046, Japan. Archaeal adaptation to higher temperatures revealed by genomic sequence of *Thermoplasma volcanium*. *Proc Natl Acad Sci U S A* **97:** (26) 14257.

Misawa K, Tajima F*. 2000. *Univ Tokyo, Dept Biol Sci, Bunkyo ku, Tokyo 113 0033, Japan. A simple method for classifying genes and a bootstrap test for classifications. *Mol Biol Evol* **17:** (12) 1879.

Mouchaty SK, Catzeflis F, Janke A, Arnason U. 2001. Baylor Coll Med, Human Genome Sequencing Ctr, 1 Baylor Plaza, Houston, Tx 77030, USA. Molecular evidence of an African phiomorpha-South American Caviomorpha clade and support for Hystricognathi based on the complete mitochondrial genome of the cane rat (*Thryonomys swinderianus*). *Mol Phylogenet Evol* **18:** (1) 127.

Mugridge NB, Morrison DA, Jakel T, Heckeroth AR, Tenter AM, Johnson AM*. 2000. *Univ Technol Sydney, Mol Parasitol Unit, Westbourne St, Gore Hill, NSW 2065, Australia. Effects of sequence alignment and structural domains of ribosomal DNA on phylogeny reconstruction for the protozoan family Sarcocystidae. *Mol Biol Evol* **17:** (12) 1842.

Nickrent DL, Parkinson CL, Palmer JD, Duff RJ. 2000. Sthn Illinois Univ, Dept Plant Biol, Carbondale, Il 62901, USA. Multigene phylogeny of land plants with special reference to bryophytes and the earliest land plants. *Mol Biol Evol* **17:** (12) 1885.

Ochman H, Jones IB. 2000. Univ Arizona, Dept Ecol & Evolutionary Biol, Tucson, Az 85721, USA. Evolutionary dynamics of full genome content in *Escherichia coli*. *EMBO J* **19:** (24) 6637.

Qiu YL, Lee J, Bernasconi-Quadroni F, Soltis DE, Soltis PS, Zanis M, Zimmer EA, Chen Z, Savolainen V, Chase MW. 2000. Univ Zurich, Inst Systemat Bot, Zollikerstr 107, CH-8008 Zurich, Switzerland. Phylogeny of basal angiosperms: Analyses of five genes from three genomes. *Int J Plant Sci* **161:** (6) S3.

Schubert M, Holland LZ, Holland ND, Jacobs DK. 2000. Scripps Inst Oceanog, Div Marine Biol, La Jolla, Ca 92093, USA. A phylogenetic tree of the Wnt genes based on all available full-length sequences, including five from the cephalochordate *Amphioxus*. *Mol Biol Evol* **17:** (12) 1896.

Seoighe C, Federspiel N, Jones T, Hansen N, Bivolarovic V, Surzycki R, Tamse R, Komp C, Hulzar L, Davis RW, Scherer S, Tait E, Shaw DJ, Harris D, Murphy L, Oliver K, Taylor K, Rajandream MA, Barrell BG, Wolfe KH*. 2000. *Univ Dublin Trinity Coll, Dept Genet, Dublin 2, Rep Ireland. Prevalence of small inversions in yeast gene order evolution. *Proc Natl Acad Sci U S A* **97:** (26) 14433.

Tracy MR, Hedges SB*. 2000. *Penn State University, Institute Mol Evolutionary Genet, 208 Mueller Lab, University Park, Pa 16802, USA. Evolutionary history of the enolase gene family. *Gene* **259:** (1-2) 129.

### 5 Comparative genomics

Chinen A, Uchiyama I, Kobayashi I*. 2000. *Univ Tokyo, Inst Med Sci, 4-6-1 Shirokanedai, Tokyo 108 8639, Japan. Comparison between *Pyrococcus horikoshii* and *Pyrococcus abyssi* genome sequence reveals linkage of restriction-modification genes with large genome polymorphisms. *Gene* **259:** (1-2) 109.

Ho TBL, Robertson BD, Taylor GM, Shaw RJ, Young DB. 2000. Imperial Coll Sch Med, Dept Infect Dis & Microbiol, Norfolk Place, London W2 1PG, England. Comparison of *Mycobacterium tuberculosis* genomes reveals frequent deletions in a 20 kb variable region in clinical isolates. *Yeast* **17:** (4) 272.

Ito N, Kakemizu M, Ito KA, Yamamoto A, Yoshida Y, Sugiyama M, Minamoto N*. 2001. *Gifu Univ, Dept Vet Publ Hlth, Yanagido 1-1, Gifu 501 1193, Japan. A comparison of complete genome sequences of the attenuated RC-HL strain of rabies virus used for production of animal vaccine in Japan, and the parental Nishigahara strain. *Microbiol Immunol* **45:** (1) 51.

Kitamura N. 2001. Yokohama City, Kowan Hosp, Dept Ophthalmol, Naka yu, 3-2-3 Shinyamashita, Yokohama, Kanagawa 231 080, Japan. Genome analysis of adenovirus type 7 and adenovirus type 11. *Jpn J Ophthalmol* **45:** (1) 22.

McClelland M, Florea L, Sanderson K, Clifton SW, Parkhill J, Churcher C, Dougan G, Wilson RK, Miller W. 2000. Sidney Kimmel Cancer Ctr, 10835 Altman Row, San Diego, Ca 92121, USA. Comparison of the *Escherichia coli* K-12 genome with sampled genomes of a *Klebsiella pneumoniae* and three *Salmonella enterica* serovars, typhimurium, typhi and paratyphi. *Nucleic Acids Res* **28:** (24) 4974.

Nekrutenko A, Li WH*. 2000. *Univ Chicago, Dept Ecol & Evolut, 940 East 57th St, Chicago, Il 60637, USA. Assessment of compositional heterogeneity within and between eukaryotic genomes. *Genome Res* **10:** (12) 1986.

Nobusato Y, Uchiyama I, Ohashi S, Kobayashi I*. 2000. *Univ Tokyo, Inst Med Sci, 4-6-1 Shirokanedai, Tokyo 108 8639, Japan. Insertion with long target duplication: A mechanism for gene mobility suggested from comparison of two related bacterial genomes. *Gene* **259:** (1-2) 99.

Postlethwait JH, Woods IG, Ngo-Hazelett P, Yan YL, Kelly PD, Chu F, Huang H, Hill-Force A, Talbot WS. 2000. Univ Oregon, Inst Neurosci, Eugene, Or 97403, USA. Zebrafish comparative genomics and the ori-

Copyright © 2001 John Wiley & Sons, Ltd. 

gins of vertebrate chromosomes. *Genome Res* **10:** (12) 1890.

Salama N, Guillemin K, McDaniel TK, Sherlock G, Tompkins L, Falkow S. 2000. Stanford Univ, Dept Microbiol & Immunol, 299 Campus Dr, Stanford, Ca 94305, USA. A whole-genome microarray reveals genetic diversity among *Helicobacter pylori* strains. *Proc Natl Acad Sci U S A* **97:** (26) 14668.

Stanchi F, Bertocco E, Toppo S, Dioguardi R, Simionati B, Cannata N, Zimbello R, Lanfranchi G, Valle G*. 2001. *Univ Padova, CRIBI Biotech Ctr, via G Colombo 3, IT-35121 Padova, Italy. Characterization of 16 novel human genes showing high similarity to yeast sequences. *Yeast* **18:** (1) 69.

Woods IG, Kelly PD, Chu F, Ngo-Hazelett P, Yan YL, Huang H, Postlethwait JH, Talbot WS*. 2000. *Stanford Univ, Dept Dev Biol, Stanford, Ca 94305, USA. A comparative map of the zebrafish genome. *Genome Res* **10:** (12) 1903.

Ze-Ze L, Tenreiro R*, Paveia H. 2000. *FCUL, Dept Biol Vegetal, Campo Grande, PT-1749016 Lisbon, Portugal. The *Oenococcus oeni* genome: Physical and genetic mapping of strain GM and comparison with the genome of a ‘divergent’ strain, PSU-1. *Microbiology* **146:** (12) 3195.

Zou D, Kaneko J, Narita S, Kamio Y*. 2000. *Tohoku Univ, Dept Mol & Cell Biol, Aoba ku, Sendai, Miyagi 981 8555, Japan. Prophage, $\phi$PV83-pro, carrying panton-valentine leukocidin genes, on the *Staphylococcus aureus* p83 chromosome: Comparative analysis of the genome structures of $\phi$PV83-pro, $\phi$PVL, $\phi$11, and other phages. *Biosci Biotechnol Biochem* **64:** (12) 2631.

### 6 Pathways, gene families and regulons

Dugas JC, Ngai J*. 2001. *Univ Calif, Dept Mol & Cell Biol, 269 Life Sci Addition, Berkeley, Ca 94720, USA. Analysis and characterization of an odorant receptor gene cluster in the zebrafish genome. *Genomics* **71:** (1) 53.

Henrissat B, Davies GJ. 2000. CNRS UMR 6098, 31 Chemin Jospeh Aiguier, FR-13402 Marseille 20, France. Glycoside hydrolases and glycosyltransferases. Families, modules, and implications for genomics. *Plant Physiol* **124:** (4) 1515.

Jakt LM, Cao L, Cheah KSE, Smith DK*. 2001. *Univ Hong Kong, Dept Biochem, Pokfulam, Hong Kong, Peoples Rep China. Assessing clusters and motifs from gene expression data. *Genome Res* **11:** (1) 112.

Koike T, Rzhetsky A*. 2000. *Columbia Univ, Columbia Genome Ctr, New York, NY 10027, USA. A graphic editor for analyzing signal-transduction pathways. *Gene* **259:** (1-2) 235.

Mold DE, Kim IF, Tsai CM, Lee D, Chang CY, Huang RCC*. 2001. *Johns Hopkins Univ, Dept Biol, 3400 Nth Charles St, Baltimore, Md 21218, USA. Cluster of genes encoding the major egg envelope protein of zebrafish. *Mol Reprod Dev* **58:** (1) 4.

Phay JE, Hussain HB, Moley JF*. 2000. *Washington Univ, Sch Med, 1 Barnes Jewish Hosp Plaza, St Louis, Mo 63110, USA. Strategy for identification of novel glucose transporter family members by using internet-based genomic databases. *Surgery* **128:** (6) 946.

Suzuki N, Nakano Y*, Yoshida Y, Nakao H, Yamashita Y, Koga T. 2000. *Kyushu Univ, Dept Prevent Dent, Fukuoka 812 8582, Japan. Genetic analysis of the gene cluster for the synthesis of serotype a-specific polysaccharide antigen in *Actinobacillus actinomycetemcomitans*. *Biochim Biophys Acta* **1517:** (1) 135.

### 7 Pharmacogenomics

Bertucci F, Houlgatte R, Benziane A, Granjeaud S, Adelaide J, Tagett R, Loriod B, Jacquemier J, Viens P, Jordan B, Birnbaum D*, Nguyen C. 2000. *INSERM U119, Lab Oncol Mol, IFR57, 27 Blvd Lei Roure, FR-13009 Marseille, France. Gene expression profiling of primary breast carcinomas using arrays of candidate genes. *Hum Mol Genet* **9:** (20) 2981.

Buetow KH, Edmonson M, MacDonald R, Clifford R, Yip P, Kelley J, Little DP, Strausberg R, Koester H, Cantor CR, Braun A. 2001. NCI, Lab Populat Genet, Div Canc Epidemiol & Genet, Bethesda, Md 20892, USA. High-throughput development and characterization of a genomewide collection of gene-based single nucleotide polymorphism markers by chip-based matrix-assisted laser desorption/ionization time-of-flight mass spectrometry. *Proc Natl Acad Sci U S A* **98:** (2) 581.

Burchiel SW, Knall CM, Davis JW, Paules RS, Boggs SE, Afshari CA. 2001. Univ New Mexico, Coll Pharm, Toxicol Program, 2502 Marble NE, Albuquerque, NM 87131, USA. Analysis of genetic and epigenetic mechanisms of toxicity: Potential roles of toxicogenomics and proteomics in toxicology. *Toxicol Sci* **59:** (2) 193.

Burczynski ME, McMillian M, Ciervo J, Li L, Parker JB, Dunn RT, Hicken S, Farr S, Johnson MD*. 2000. *Robert Wood Johnson Pharmaceut Res Inst, Drug Safety Evaluat, POB 300, Route 202, Raritan, NJ 08869, USA. Toxicogenomics-based discrimination of toxic mechanism in HepG2 human hepatoma cells. *Toxicol Sci* **58:** (2) 399.

Galloway AM, Allalunis-Turner J*. 2000. *Cross Cancer Inst, Dept Oncol, 11560 Univ Ave, Edmonton, Alberta, Canada T6G 1Z2. cDNA expression array analysis of DNA repair genes in human glioma cells that lack or express DNA-PK. *Radiat Res* **154:** (6) 609.

Huang HT, Colella S, Kurrer M, Yonekawa Y, Kleihues P, Ohgaki H*. 2000. *Int Agcy Res Cancer, Unit Mol Pathol, 150 Cours Albert Thomas, FR-69372 Lyon 08, France. Gene expression profiling of low-grade diffuse astrocytomas by cDNA arrays. *Cancer Res* **60:** (24) 6868.

Lau WY, Lai PBS, Leung MF, Leung BCS, Wong N, Chen G, Leung TWT, Liew CT. 2000. Chinese Univ, Prince of Wales Hosp, Dept Surg, Shatin, Hong Kong, Peoples Rep China. Differential gene expression of hepatocellular carcinoma using cDNA microarray analysis. *Oncol Res* **12:** (2) 59.

Lewohl JM, Wang L, Miles MF, Zhang L, Dodd PR, Harris RA*. 2000. *Univ Texas, Waggoner Ctr Alcohol & Addict Res, Mol Biol Bldg, Austin, Tx 78712, USA. Gene expression in human alcoholism: Microarray analysis of frontal cortex. *Alcohol Clin Exp Res* **24:** (12) 1873.

Ljubimova JY, Khazenzon NM, Chen ZT, Neyman YI, Turner L, Riedinger MS, Black KL. 2001. Cedars Sinai Med Ctr, Maxine Dunitz Neurosurg Inst, 8631 West 3rd St, Los Angeles, Ca 90048, USA. Gene expression abnormalities in human glial tumors identified by gene array. *Int J Oncol* **18:** (2) 287.

Olesen C, Hansen C, Bendsen E, Byskov AG, Schwinger E, Lopez-Pajares I, Jensen PKA, Kristoffersson U, Schubert R, Van Assche E, Wahlstroem J, Lespinasse J, Tommerup N*. 2001. *Univ Copenhagen, Dept Med Genet, Blegdamsvej 3, DK-2200 Copenhagen, Denmark. Identification of human candidate genes for male infertility by digital differential display. *Mol Hum Reprod* **7:** (1) 11.

Sallinen SL, Sallinen PK, Haapasalo HK, Helin HJ, Helen PT, Schraml P, Kallioniemi OP, Kononen J*. 2000. *NIH/NHGRI, Canc Genet Lab, 49 Convent Dr, MSC 4470, Room 4A24, Bethesda, Md 20892, USA. Identification of differentially expressed genes in human gliomas by DNA microarray and tissue chip techniques. *Cancer Res* **60:** (23) 6617.

Stern LE, Erwin CR, Falcone RA, Huang FS, Kemp CJ, Williams JL, Warner BW*. 2001. *Univ Cincinnati, Children’s Hosp Med Ctr, Div Pediat Surg, 3333 Burnet Ave, Cincinnati, Oh 45229, USA. cDNA microarray analysis of adapting bowel after intestinal resection. *J Pediatr Surg* **36:** (1) 190.

Tanaka T, Nishimura Y, Tsunoda H, Naka M. 2000. Mie Univ, Dept Mol & Cellular Pharmacol, Tsu, Mie 514 8507, Japan. Pharmacogenomics and therapeutic target validation in cerebral vasospasm. *J Cardiovasc Pharmacol* **36:** (6 Suppl 2) S1.

Vercoutter-Edouart AS, Lemoine J, Le Bourhis X, Louis H, Boilly B, Nurcombe V, Revillion F, Peyrat JP, Hondermarck H*. 2001. *Univ Lille 1, UPRES EA Biol Dev 1033, Bat SN3, FR-59650 Villeneuve d’Ascq, France. Proteomic analysis reveals that 14-3-3$\sigma$ is down-regulated in human breast cancer cells. *Cancer Res* **61:** (1) 76.

Wolf M, El Rifai W, Tarkkanen M, Kononen J, Serra M, Eriksen EF, Elomaa I, Kallioniemi A, Kallioniemi OP, Knuutila S*. 2000. *Univ Helsinki, Haartman Inst, Dept Med Genet, Helsinki, Finland. Novel findings in gene expression detected in human osteosarcoma by cDNA microarray. *Cancer Genet Cytogenet* **123:** (2) 128.

### 8 Large-scale mutagenesis programmes

Mollapour M, Piper PW*. 2001. *UCL, Dept Biochem & Molec Biol, London WC1E 6BT, England. Targeted gene deletion in *Zygosaccharomyces bailii*. *Yeast* **18:** (2) 173.

Copyright © 2001 John Wiley & Sons, Ltd. 

Muren E, Oyen M, Barmark G, Ronne H*. 2001. *Swedish Univ Agric Sci, Dept Plant Biol, Uppsala Genet Ctr, Box 7080, SE-750 07 Uppsala, Sweden. Identification of yeast deletion strains that are hypersensitive to brefeldin A or monensin, two drugs that affect intracellular transport. *Yeast* **18:** (2) 163.

### 9 Functional genomics

Abdrakhmanov I, Lodygin D, Geroth P, Arakawa H, Law A, Plachy J, Korn B, Buerstedde JM*. 2000. *Heinrich Pette Inst Expt Virol & Immunol, Dept Cellular Immunol, DE-20251 Hamburg, Germany. A large database of chicken bursal ESTs as a resource for the analysis of vertebrate gene function. *Genome Res* **10:** (12) 2062.

Aguan K, Carvajal JA, Thompson LP, Weiner CP. 2000. Univ Maryland, Dept Obstet Gynecol & Reprod Sci, Bresseler Res Bldg 11-048, Baltimore, Md 21201, USA. Application of a functional genomics approach to identify differentially expressed genes in human myometrium during pregnancy and labour. *Mol Hum Reprod* **6:** (12) 1141.

Bulger M, Bender MA, Van Doorninck JH, Wertman B, Farrell CM, Felsenfeld G, Groudine M*, Hardison R. 2000. *Fred Hutchinson Cancer Res Ctr, 1100 Fairview Ave Nth, Seattle, Wa 98109, USA. Comparative structural and functional analysis of the olfactory receptor genes flanking the human and mouse β-globin gene clusters. *Proc Natl Acad Sci U S A* **97:** (26) 14560.

Galperin MY. 2001. NIH/Nat Ctr Biotechnol Inform, Nat Library Med, Bethesda, Md 20894, USA. Conserved 'hypothetical' proteins: New hints and new puzzles (Conference Paper). *Comp Funct Genom* **2:** (1) 14.

Grammer TC, Liu KJ, Mariani FV, Harland RM*. 2000. *Univ Calif, Dept Mol & Cell Biol, 401 Barker Hall, Berkeley, Ca 94720, USA. Use of large-scale expression cloning screens in the *Xenopus laevis* tadpole to identify gene function. *Dev Biol* **228:** (2) 197.

Gurvitz A, Langer S, Piskacek M, Hamilton B, Ruis H, Hartig A. 2000. Univ Vienna, Inst Biochem & Molek Zellbiol, Vienna Bioctr, Dr Bohrgasse 9, AU-1030 Vienna, Austria. Predicting the function and subcellular location of *Caenorhabditis elegans* proteins similar to *Saccharomyces cerevisiae* β-oxidation enzymes. *Yeast* **17:** (3) 188.

Harwood CR, Crawshaw SG, Wipat A. 2001. Univ Newcastle upon Tyne, Dept Microbiol & Immunol, Framlington Place, Newcastle-upon-Tyne NE2 4HH, England. From genome to function: Systematic analysis of the soil bacterium *Bacillus subtilis* (Conference Paper). *Comp Funct Genom* **2:** (1) 22.

Kawai J, Shinagawa A, Shibata K, Yoshino M, Itoh M, Ishii Y, Arakawa T, Hara A, Fukunishi Y, Konno H *et al*. 2001. Yokohama Inst, RIKEN Genome Sci Ctr, Lab Genome Exploration, Res Grp, Tsurumi ku, 1-7-22 Suehiro cho, Kanagawa 230 0045, Japan. Functional annotation of a full-length mouse cDNA collection. *Nature* **409:** (6821) 685.

Kuhn KM, De Risi JL, Brown PO*, Sarnow P. 2001. *Stanford Univ, Dept Microbiol & Immunol, Stanford, Ca 94305, USA. Global and specific translational regulation in the genomic response of *Saccharomyces cerevisiae* to a rapid transfer from a fermentable to a nonfermentable carbon source. *Mol Cell Biol* **21:** (3) 916.

Maneu V, Roig P, Gozalbo D*. 2000. *Univ Valencia, Dept Microbiol & Ecol, Avda Vicent Andres Estelles s/n, ES-46100 Valencia, Spain. Complementation of *Saccharomyces cerevisiae* mutations in genes involved in translation and protein folding (*EFB1* and *SSB1*) with *Candida albicans* cloned genes. *Res Microbiol* **151:** (9) 739.

Moller S, Kriventseva EV, Apweiler R. 2000. European Bioinformat Inst, Cambridge, England. A collection of well characterised integral membrane proteins. *Bioinformatics* **16:** (12) 1159.

Ton C, Hwang DM, Dempsey AA, Tang HC, Yoon J, Lim M, Mably JD, Fishman MC, Liew CC*. 2000. *Univ Toronto, Dept Lab Med & Pathobiol, 100 Coll St, Toronto, Ontario, Canada M5G 1L5. Identification, characterization, and mapping of expressed sequence tags from an embryonic zebrafish heart cDNA library. *Genome Res* **10:** (12) 1915.

Tsukamoto T, Hashiguchi N, Janicki SM, Tumbar T, Belmont AS, Spector DL*. 2000. *Cold Spring Harbor Lab, 1 Bungtown Rd, Cold Spring Harbor, NY 11724, USA. Visualization of gene activity in living cells. *Nat Cell Biol* **2:** (12) 871.

Volkert MR, Elliott NA, Housman DE. 2000. Univ Massachusetts, Dept Mol Genet & Microbiol, Worcester, Ma 01655, USA. Functional genomics reveals a family of eukaryotic oxidation protection genes. *Proc Natl Acad Sci U S A* **97:** (26) 14530.

White JA, Todd T, Newman T, Focks N, Girke T, Dellarduya OM, Jaworski JG, Ohlrogge JB, Benning C*. 2000. *State Univ, Dept Biochem & Mol Biol, East Lansing, Mi 48824, USA. A new set of *Arabidopsis* expressed sequence tags from developing seeds. The metabolic pathway from carbohydrates to seed oil. *Plant Physiol* **124:** (4) 1582.

### 10 Transcriptomics

Andrews J, Bouffard GG, Cheadle C, Lu JN, Becker KG, Oliver B*. 2000. *NIH/NIDDKD, Cellular & Dev Biol Lab, Bethesda, Md 20892, USA. Gene discovery using computational and microarray analysis of transcription in the *Drosophila melanogaster* tests. *Genome Res* **10:** (12) 2030.

Bartosiewicz M, Penn S, Buckpitt A. 2001. Univ Calif, Dept Mol Biosci, Haring Hall, Davis, Ca 95616, USA. Applications of gene arrays in environmental toxicology: Fingerprints of gene regulation associated with cadmium chloride, benzo(a)pyrene, and trichloroethylene. *Environ Health Perspect* **109:** (1) 71.

Callow MJ, Dudoit S, Gong EL, Speed TP, Rubin EM. 2000. Univ Calif, Lawrence Berkeley Lab, Dept Genome Sci, Berkeley, Ca 94720, USA. Microarray expression profiling identifies genes with altered expression in HDL-deficient mice. *Genome Res* **10:** (12) 2022.

Chrast R, Scott HS, Papasavvas MP, Rossier C, Antonarakis ES, Barras C, Davisson MT, Schmidt C, Estivill X, Dierssen M, Pritchard M, Antonarkis SE*. 2000. *Univ Geneva, Div Med Genet, CH-1211 Geneva, Switzerland. The mouse brain transcriptome by SAGE: Differences in gene expression between P30 brains of the partial trisomy 16 mouse model of Down syndrome (Ts65Dn) and normals. *Genome Res* **10:** (12) 2006.

Douglas KR, Camper SA*. 2000. *Univ Michigan, Dept Human Genet, 4301 Med Sci Res Bldg 3, Ann Arbor, Mi 48109, USA. Partial transcriptome of the developing pituitary gland. *Genomics* **70:** (3) 335.

Farlow DN, Vansant G, Cameron AA, Chang J, Khoh-Reiter S, Pham NL, Wu W, Sagara Y, Nicholls JG, Carlo DJ, Ill CR*. 2000. *Immune Response Corp, 5935 Darwin Court, Carlsbad, Ca 92008, USA. Gene expression monitoring for gene discovery in models of peripheral and central nervous system differentiation, regeneration, and trauma. *J Cell Biochem* **80:** (2) 171.

Girke T, Todd J, Ruuska S, White J, Benning C, Ohlrogge J*. 2000. *Michigan State Univ, Dept Bot & Plant Pathol, East Lansing, Mi 48824, USA. Microarray analysis of developing *Arabidopsis* seeds. *Plant Physiol* **124:** (4) 1570.

Gmuender H, Kuratli K, Di Padova K, Gray CP, Keck W, Evers S. 2001. F Hoffmann La Roche & Co Ltd, Div Pharmaceut, CH-4070 Basel, Switzerland. Gene expression changes triggered by exposure of *Haemophilus influenzae* to novobiocin or ciprofloxacin: Combined transcription and translation analysis. *Genome Res* **11:** (1) 28.

Kenzelmann M, Muhlemann K. 2000. Univ Bern, Inst Med Microbiol, Friedbuhlstr 51, CH-3010 Bern, Switzerland. Transcriptome analysis of fibroblast cells immediate-early after human cytomegalovirus infection. *J Mol Biol* **304:** (5) 741.

Maleck K, Levine A, Eulgem T, Morgan A, Schmid J, Lawton KA, Dangl JL*, Dietrich RA. 2000. *Univ Nth Carolina, Dept Biol, CB 3280, Chapel Hill, NC 27599, USA. The transcriptome of *Arabidopsis thaliana* during systemic acquired resistance. *Nat Genet* **26:** (4) 403.

Nadadur SS, Schladweiler MCJ, Kodavanti UP. 2000. US/EPA, Natl Hlth & Environm Effects Res Lab, Pulm Toxicol Branch, Res Triangle Park, NC 27711, USA. A pulmonary rat gene array for screening altered expression profiles in air pollutant-induced lung injury. *Inhal Toxicol* **12:** (12) 1239.

Ogawa N, De Risi J, Brown PO*. 2000. *Stanford Univ, Howard Med Inst, Dept Biochem, Stanford, Ca 94305, USA. New components of a system for phosphate accumulation and polyphosphate metabolism in *Saccharomyces cerevisiae* revealed by genomic expression analysis. *Mol Biol Cell* **11:** (12) 4309.

Primig M, Williams RM, Winzeler EA, Tevzadze GG, Conway AR, Hwang SY, Davis RW, Esposito RE*. 2000. *Univ Chicago, Dept Mol Genet & Cell Biol, Chicago, Il 60637, USA. The core meiotic transcriptome in budding yeasts. *Nat Genet* **26:** (4) 415.

Selinger DW, Cheung KJ, Mei R, Johansson EM, Richmond CS, Blattner FR, Lockhart DJ, Church GM*. 2000. *Harvard Univ, Dept Genet, 200 Longwood Ave, Boston, Ma 02115, USA. RNA expression analysis using a 30 base pair resolution *Escherichia coli* genome array. *Nat Biotechnol* **18:** (12) 1262.

Copyright © 2001 John Wiley & Sons, Ltd. 

Shi NY, Boado RJ, Pardridge WM*. 2000. *UCLA, Dept Med, Los Angeles, Ca 90095, USA. Antisense imaging of gene expression in the brain *in vivo*. *Proc Natl Acad Sci U S A* **97:** (26) 14709.
Stekel DJ, Git Y, Falciani F. 2000. Oxford Gene Technol, Littlemore Pk, Oxford OX4 4SS, England. The comparison of gene expression from multiple cDNA libraries. *Genome Res* **10:** (12) 2055.
Van Kampen AHC, Van Schaik BDC, Pauws E, Michiels EMC, Ruijter JM, Caron HN, Versteeg R, Heisterkamp SH, Leunissen JAM, Baas F, Van der Mee M. 2000. Univ Amsterdam, Bioinformat Lab, Meibergdreef 9, NL-1100 AZ Amsterdam, The Netherlands. USAGE: A web-based approach towards the analysis of SAGE data. *Bioinformatics* **16:** (10) 899.
Vertegaal ACO, Kuiperij HB, Van Laar T, Scharnhorst V, Van der Eb AJ, Zantema A*. 2000. *Leiden Univ, Dept Mol Cell Biol, Wassenaarsweg 72, NL-2333 AL Leiden, The Netherlands. cDNA microarray identification of a gene differentially expressed in adenovirus type 5-*versus* type 12-transformed cells. *FEBS Lett* **487:** (2) 151.
Wei Y, Lee JM, Richmond C, Blattner FR, Rafalski JA, La Rossa RA*. 2001. *DuPont Co Inc, Cent Res & Dev Biochem Sci & Engn, Expt Stn, POB 80173, Wilmington, De 19880, USA. High-density microarray-mediated gene expression profiling of *Escherichia coli*. *J Bacteriol* **183:** (2) 545.
Zhu T, Wang X*. 2000. *Novartis Agr Discovery Inst Inc, 3115 Merryfield Row, San Diego, Ca 92121, USA. Large-scale profiling of the *Arabidopsis* transcriptome. *Plant Physiol* **124:** (4) 1472.

### 11 Proteomics

Albala JS, Franke K, McConnell IR, Pak KL, Folta PA, Karlak B, Rubinfeld B, Davies AH, Lennon GG, Clark R. 2000. Lawrence Livermore Natl Lab, Biol & Biotechnol Res Program, 7000 East Ave, Livermore, Ca 94550, USA. From genes to proteins: High-throughput expression and purification of the human proteome. *J Cell Biochem* **80:** (2) 187.
Betts JC, Dodson P, Quan S, Lewis AP, Thomas PJ, Duncan K, McAdam RA. 2000. Glaxo Wellcome R&D Ltd, Med Res Ctr, Stevenage SG1 2NY, England. Comparison of the proteome of *Mycobacterium tuberculosis* strain H37Rv with clinical isolate CDC 1551. *Microbiology* **146:** (12) 3205.
Garin J, Diez R, Kieffer S, Dermine JF, Duclos S, Gagnon E, Sadoul R, Rondeau C, Desjardins M*. 2001. *Univ Montreal, Dept Pathol & Biol Cellulaire, CP 6128, Succ Ctr-Ville, Montreal, Quebec, Canada H3C 3J7. The phagosome proteome: Insight into phagosome functions. *J Cell Biol* **152:** (1) 165.
Gerner C, Frohwein U, Gotzmann J, Bayer E, Gelbmann D, Bursch W, Schulte-Hermann R. 2000. Univ Vienna, Cancer Res Inst, Borschkegasse 8A, AU-1090 Vienna, Austria. The Fas-induced apoptosis analyzed by high throughput proteome analysis. *J Biol Chem* **275:** (50) 39018.
Han MJ, Yoon SS, Lee SY*. 2001. *KAIST, Metab & Biomol Engn Natl Res Lab, 373-1 Kusong dong, Taejon 305701, South Korea. Proteome analysis of metabolically engineered *Escherichia coli* producing poly(3-hydroxybutyrate). *J Bacteriol* **183:** (1) 301.
Karlberg O, Canback B, Kurland CG, Andersson SGE*. 2000. *Uppsala Univ, Dept Molec Evolution, Norbyvagen 18C, SE-75234 Uppsala, Sweden. The dual origin of the yeast mitochondrial proteome. *Yeast* **17:** (3) 170.
Lewis TS, Hunt JB, Aveline LD, Jonscher KR, Louie DF, Yeh JM, Nahreini TS, Resing KA, Ahn NG*. 2000. *Univ Colorado, Dept Chem & Biochem, Boulder, Co 80309, USA. Identification of novel MAP kinase pathway signaling targets by functional proteomics and mass spectrometry. *Mol Cell* **6:** (6) 1343.
Matte-Tailliez O, Zivanovic Y, Forterre P. 2000. Univ Paris Sud, Inst Genet & Microbiol, UMR C8621, FR-91405 Orsay, France. Mining archaeal proteomes for eukaryotic proteins with novel functions: The PACE case. *Trends Genet* **16:** (12) 533.
Oosthuizen MC, Steyn B, Lindsay D, Brozel VS*, Von Holy A. 2001. *Univ Pretoria, Dept Microbiol & Plant Pathol, Lab Biofilm Physiol, ZA-0001 Pretoria, Rep Sth Africa. Novel method for the proteomic investigation of a dairy-associated *Bacillus cereus* biofilm. *FEMS Microbiol Lett* **194:** (1) 47.
Rain JC, Selig L, De Reuse H, Battaglia V, Reverdy C, Simon S, Lenzen G, Petel F, Wojcik J, Schachter V, Chemama Y, Labigne A, Legrain P*. 2001. *Hybrigen SA, 180 Ave Daumesnil, FR-75012 Paris, France. The protein-protein interaction map of *Helicobacter pylori*. *Nature* **409:** (6817) 211.
Schwikowski B, Uetz P, Fields S. 2000. Inst Syst Biol, 4225 Roosevelt Way NE, Suite 200, Seattle, Wa 98105, USA. A network of protein-protein interactions in yeast. *Nat Biotechnol* **18:** (12) 1257.
Wu CC, Taylor RS, Lane DR, Ladinsky MS, Weisz JA, Howell KE*. 2000. *Univ Colorado, Hlth Sci Ctr, Dept Cellular & Struct Biol, Denver, Co 80262, USA. GMx33: A novel family of trans-Golgi proteins identified by proteomics. *Traffic* **1:** (12) 963.

### 12 Protein structural genomics

Chen WB, Laidig KE, Park Y, Park K, Yates JR, Lamont RJ, Hackett M*. 2001. *Univ Washington, Dept Med Chem, Box 357610, Seattle, Wa 98195, USA. Searching the *Porphyromonas gingivalis* genome with peptide fragmentation mass spectra. *Analyst* **126:** (1) 52.
Gaucher EA, Miyamoto MM, Benner SA. 2001. Univ Florida, Dept Chem, Benners Lab, Leigh Hall 440, Gainesville, Fl 32611, USA. Function-structure analysis of proteins using covarion-based evolutionary approaches: Elongation factors. *Proc Natl Acad Sci U S A* **98:** (2) 548.
Irving JA, Whisstock JC, Lesk AM*. 2001. *Univ Cambridge, Sch Clin, Cambridge Inst Med Res, Dept Haematol, Cambridge CB2 2QH, England. Protein structural alignments and functional genomics. *Protein-Struct Funct Genet* **42:** (3) 378.
Kawabata T, Arisaka F, Nishikawa K*. 2000. *Natl Inst Genet, Ctr Informat Biol, 1111 Yata, Mishima, Shizuoka 411 854, Japan. Structural/functional assignment of unknown bacteriophage T4 proteins by iterative database searches. *Gene* **259:** (1-2) 223.

### 13 Metabolomics

Raamsdonk LM, Teusink B, Broadhurst D, Zhang NS, Hayes A, Walsh MC, Berden JA, Brindle KM, Kell DB, Rowland JJ, Westerhoff HV, Van Dam K, Oliver SG*. 2001. *Univ Manchester, Sch Biol Sci, 2-205 Stopford Bldg, Oxford Rd, Manchester M13 9PT, England. A functional genomics strategy that uses metabolome data to reveal the phenotype of silent mutations. *Nat Biotechnol* **19:** (1) 45.

### 14 Genomic approaches to development

Itoh JI, Kitano H, Matsuoka M, Nagato Y*. 2000. *Univ Tokyo, Grad Sci Agr & Life Sci, Tokyo 113 8657, Japan. *SHOOT ORGANIZATION* genes regulate shoot apical meristem organization and the pattern of leaf primordium initiation in rice. *Plant Cell* **12:** (11) 2161.
Jernvall J, Keranen SVE, Thesleff I. 2000. Univ Helsinki, Viikki Bioctr, Inst Biotechnol, POB 56, Viikinkaari, FI-00014 Helsinki, Finland. Evolutionary modification of development in mammalian teeth: Quantifying gene expression patterns and topography. *Proc Natl Acad Sci U S A* **97:** (26) 14444.
Nasevicius A, Larson J, Ekker SC*. 2000. *Univ Minnesota, Dept Genet, Cell Biol & Dev, Beckman Ctr Transposon Res, 6-160 Jackson Hall, 321 Church St SE, Minneapolis, Mn 55455, USA. Distinct requirements for zebrafish angiogenesis revealed by a *VEGF-A* morphant. *Yeast* **17:** (4) 294.
Perz-Edwards A, Hardison NL, Linney E. 2001. Duke Univ, Med Ctr, Dept Cell Biol, Durham, NC 27710, USA. Retinoic acid-mediated gene expression in transgenic reporter zebrafish. *Dev Biol* **229:** (1) 89.
Rast JP, Amore G, Calestani C, Livi CB, Ransick A, Davidson EH*. 2000. *Calif Technol Inst, Div Biol, Pasadena, Ca 91125, USA. Recovery of developmentally defined gene sets from high-density cDNA macroarrays. *Dev Biol* **228:** (2) 270.
Schiefelbein JW. 2000. Univ Michigan, Dept Biol, 830 Nth Univ Ave, Ann Arbor, Mi 48109, USA. Constructing a plant cell. The genetic control of root hair development. *Plant Physiol* **124:** (4) 1525.

### 15 Technological advances

Al-Taher A, Bashein A, Nolan T, Hollingsworth M, Brady G*. 2000. *Univ Manchester, Sch Biol Sci, G.38 Stopford Bldg, Oxford Rd, Manchester M13 9PT, England. Global cDNA amplification combined

Copyright © 2001 John Wiley & Sons, Ltd. 

with real-time RT-PCR: Accurate quantification of multiple human potassium channel genes at the single cell level. *Yeast* **17:** (3) 201.

Albargheuthi MN, Barron AE*. 2000. *Northwestern Univ, Dept Chem Engn, 2145 Sheridan Rd, Evanston, Il 60208, USA. Polymeric matrices for DNA sequencing by capillary electrophoresis. *Electrophoresis* **21:** (18) 4096.

Biyani M, Nishigaki K*. 2001. *Saitama Univ, Dept Funct Mat Sci, 255 Shimo Okubo, Urawa, Saitama 338 8570, Japan. Hundredfold productivity of genome analysis by introduction of microtemperature-gradient gel electrophoresis. *Electrophoresis* **22:** (1) 23.

Buchholz BA, Doherty EAS, Alberghouthi MN, Bogdan FM, Zahn JM, Barron AE*. 2001. *Northwestern Univ, Dept Chem Engn, Evanston, Il 60208, USA. Microchannel DNA sequencing matrices with a thermally controlled “viscosity switch”. *Anal Chem* **73:** (2) 157.

Dauter Z, Li M, Wlodawer A. 2001. NCI, Program Struct Biol, Bldg 725A-X9, Upton, NY 11973, USA. Practical experience with the use of halides for phasing macromolecular structures: A powerful tool for structural genomics. *Acta Crystallogr D Biol Crystallogr* **57:** (2) 239.

Eguchi Y, Eguchi T*. 2001. *Univ Ryukyus, Fac Med, Dept Biochem, Okinawa 903 0215, Japan. RNA electrophoretic mobility shift assay using a fluorescent DNA sequencer. *Biotechnol Lett* **23:** (2) 91.

Kadota K, Miki R, Bono H, Shimizu K, Okazaki Y*, Hayashizaki Y. 2001. *RIKEN Yokohama Inst, Genome Sci Ctr, Lab Genome Explorat Res Grp, Tsurumi ku, Kanagawa 230 004, Japan. Preprocessing implementation for microarray (PRIM): An efficient method for processing cDNA microarray data. *Physiol Genomics* **4:** (3) 183.

Kim S, Zeller K, Dang CV, Sandgren EP, Lee LA*. 2001. *Johns Hopkins Univ, Dept Med, 720 Rutland Ave, Baltimore, Md 21205, USA. A strategy to identify differentially expressed genes using representational difference analysis and cDNA arrays. *Anal Biochem* **288:** (2) 141.

Luo LY, Diamandis EP*. 2000. *Mt Sinai Hosp, Dept Pathol & Lab Med, 600 Univ Ave, Toronto, Ontario, Canada M5G 1X5. Preliminary examination of time-resolved fluorometry for protein array applications. *Luminescence* **15:** (6) 409.

Nelson BP, Grimsrud TE, Liles MR, Goodman RM, Corn RM*. 2001. *Univ Wisconsin, Dept Chem, 1101 Univ Ave, Madison, Wi 53706, USA. Surface plasmon resonance imaging measurements of DNA and RNA hybridization adsorption onto DNA microarrays. *Anal Chem* **73:** (1) 1.

Schadt EE, Li C, Su C, Wong WH. 2000. UCLA, Dept Biomath, Los Angeles, Ca 90095, USA. Analyzing high-density oligonucleotide gene expression array data. *J Cell Biochem* **80:** (2) 192.

Taniguchi M, Miura K, Iwao H, Yamanaka S*. 2001. *Nara Inst Sci & Technol, Res & Educ Ctr Genet Informat, Nara 630 0101, Japan. Quantitative assessment of DNA microarrays: Comparison with Northern blot analyses. *Genomics* **71:** (1) 34.

Tenenbaum SA, Carson CC, Lager PJ, Keene JD*. 2000. *Duke Univ, Dept Microbiol, Res Dr, Durham, NC 27710, USA. Identifying mRNA subsets in messenger ribonucleoprotein complexes by using cDNA arrays. *Proc Natl Acad Sci U S A* **97:** (26) 14085.

Towery RB, Fawcett NC*, Zhang P, Evans JA. 2001. *Univ Sthn Mississippi, Dept Chem & Biochem, Hattiesburg, Ms 39406, USA. Genomic DNA hybridizes with the same rate constant on the QCM biosensor as in homogeneous solution. *Biosens Bioelectron* **16:** (1-2) 1.

Waddell E, Wang Y, Stryjewski W, McWhorter S, Henry AC, Evans D, McCarley RL*, Soper SA. 2000. *NIST, Gaithersburg, Md 20899, USA. High-resolution near-infrared imaging of DNA microarrays with time-resolved acquisition of fluorescence lifetimes. *Anal Chem* **72:** (24) 5907.

Wildsmith SE, Archer GE, Winkley AJ, Lane PW, Bugelski PJ. 2001. SmithKline Beecham Pharmaceut, Safety Assessment, Welwyn Garden City AL6 9AR, England. Maximization of signal derived from cDNA microarrays. *Biotechniques* **30:** (1) 202.

Wisman E, Ohlrogge J. 2000. Michigan State Univ, East Lansing, Mi 48824, USA. *Arabidopsis* microarray service facilities. *Plant Physiol* **124:** (4) 1468.

### 16 Bioinformatics

Anastassiou D. 2000. Columbia Univ, Dept Elect Engn, 500 West 120th St, Mail Code 4712, New York, NY 10027, USA. Frequency-domain analysis of biomolecular sequences. *Bioinformatics* **16:** (12) 1073.

Apweiler R, Attwood TK, Bairoch A, Bateman A, Birney E, Biswas M, Bucher P, Cerutti L, Corpet F, Croning MDR *et al*. 2000. European Bioinformat Inst, EMBL Outstn, Wellcome Trust, Genome Campus, Cambridge, England. InterPro: An integrated documentation resource for protein families, domains and functional sites. *Bioinformatics* **16:** (12) 1145.

Baldi P, Baisnee PF. 2000. Univ Calif Irvine, Dept Information & Comp Sci, Irvine, Ca 92697, USA. Sequence analysis by additive scales: DNA structure for sequences and repeats of all lengths. *Bioinformatics* **16:** (10) 865.

Bedell JA, Korf I, Gish W*. 2000. *Washington Univ, Genome Sequencing Ctr, St Louis, Mo 63108, USA. *MaskerAid*: A performance enhancement to RepeatMasker. *Bioinformatics* **16:** (11) 1040.

Beissbarth T, Fellenberg K, Brors B, Arribas-Prat R, Boer JM, Hauser NC, Scheideler M, Hoheisel JD, Schutz G, Poustka A, Vingron M* 2000. *Deutsch Krebsforchungszentrum, Abt Theoret Bioinformat, Inf 280, DE-69120 Heidelberg, Germany. Processing and quality control of DNA array hybridization data. *Bioinformatics* **16:** (11) 1014.

Bingham J, Plowman GD, Sudarsanam S*. 2000. *Sugen Inc, 230 East Grand Ave, Sth San Francisco, Ca 94080, USA. Informatics issues in large-scale sequence analysis: Elucidating the protein kinases of *C. elegans*. *J Cell Biochem* **80:** (2) 181.

Boone M, Upton C*. 2000. *Univ Victoria, Dept Biochem & Microbiol, POB 3055, Victoria, Brit Columbia, Canada V8W 3P6. BLAST Search Updater: A notification system for new database matches. *Bioinformatics* **16:** (11) 1054.

Bouton CMLS, Pevsner J*. 2000. *Johns Hopkins Sch Med, Dept Neurosci, Baltimore, Md 21205, USA. DRAGON: Database Referencing of Array Genes Online. *Bioinformatics* **16:** (11) 1038.

Campagne F. 2000. CUNY Mt Sinai Sch Med, Dept Physiol & Biophys, Box 1218, 1 Gustave L Levy Pl, New York, NY 10029, USA. Clustalnet: The joining of Clustal and CORBA. *Bioinformatics* **16:** (7) 606.

Choe W, Ersoy OK, Bina M. 2000. Purdue Univ, Sch Elect & Comp Engn, West Lafayette, In 47907, USA. Neural network schemes for detecting rare events in human genomic DNA. *Bioinformatics* **16:** (12) 1062.

Choi JH, Jung HY, Kim HS, Cho HG. 2000. Pusan Natl Univ, Dept Comp Sci, Pusan 609735, South Korea. PhyloDraw: A phylogenetic tree drawing system. *Bioinformatics* **16:** (11) 1056.

Furey TS, Cristianini N, Duffy N, Bednarski DW, Schummer M, Haussler D. 2000. Univ Calif, Dept Comp Sci, Santa Cruz, Ca 95064, USA. Support vector machine classification and validation of cancer tissue samples using microarray expression data. *Bioinformatics* **16:** (10) 906.

Hao BL, Xie HM, Yu ZG, Chen GY. 2000. Acad Sinica, Inst Theoret Phys, POB 2735, CN-100080 Beijing, Peoples Rep China. Factorizable language: From dynamics to bacterial complete genomes. *Physica A* **288:** (1-4) 10.

Herwig R, Schmitt AO, Steinfath M, O’Brien J, Seidel H, Meier-Ewert S, Lehrach H, Radelof U. 2000. Max-Planck-Inst Mol Genet, Ihnestr 73, DE-14195 Berlin, Germany. Information theoretical probe selection for hybridisation experiments. *Bioinformatics* **16:** (10) 890.

Karp PD. 2001. SRI Int, Bioinformatics Res Grp, 333 Ravenswood Ave, Menlo Park, Ca 94025, USA. Many Genbank entries for complete microbial genomes violate the Genbank standard (Conference Paper). *Comp Funct Genom* **2:** (1) 25.

Kersey P, Hermjakob H, Apweiler R. 2000. European Bioinformat Inst, EMBL Outstn Hinxton, Wellcome Trust Genome Campus, Hinxton CB10 1SD, England. VARSPLIC: Alternatively-spliced protein sequences derived from SWISS-PROT and TrEMBL. *Bioinformatics* **16:** (11) 1048.

King RD, Karwath A, Clare A, Dehaspe L. 2000. Univ Wales, Dept Computer Sci, Penglais, Aberystwyth SY23 3DB, Wales. Accurate prediction of protein functional class from sequence in the *Mycobacterium tuberculosis* and *Escherichia coli* genomes using data mining. *Yeast* **17:** (4) 283.

Korf I, Gish W. 2000. Washington Univ, Sch Med, Genome Sequencing Ctr, St Louis, Mo 63108, USA. *MPBLAST*: Improved BLAST performance with multiplexed queries. *Bioinformatics* **16:** (11) 1052.

Krauthammer M, Rzhetsky A, Morozov P, Friedman C. 2000. Columbia Univ, Dept Med Informat, New York, NY 10027, USA. Using BLAST for identifying gene and protein names in journal articles. *Gene* **259:** (1-2) 245.

Li C, Wong WH*. 2001. *Harvard Univ, Dept Biostat, 655 Huntington

Ave, Boston, Ma 02115, USA. Model-based analysis of oligonucleotide arrays: Expression index computation and outlier detection. *Proc Natl Acad Sci U S A* **98:** (1) 31.

Li WZ, Pio F, Pawlowski K, Godzik A*. 2000. *Burnham Inst, La Jolla, Ca 92037, USA. Saturated BLAST: An automated multiple intermediate sequence search used to detect distant homology. *Bioinformatics* **16:** (12) 1105.

Liao BR, Hale W, Epstein CB, Butow RA, Garner HR. 2000. UT SW Med Ctr, Ctr Biomed Invent, 5323 Harry Hines Blvd, Dallas, Tx 75390, USA. MAD: A suite of tools for microarray data management and processing. *Bioinformatics* **16:** (10) 946.

Lio P, Vannucci M. 2000. Univ Cambridge, Dept Zool, Downing St, Cambridge CB2 3EH, England. Finding pathogenicity islands and gene transfer events in genome data. *Bioinformatics* **16:** (10) 932.

Man MZ, Wang XN, Wang YX. 2000. PGRD, Biostatistics, 2800 Plymouth Rd, Ann Arbor, Mi 48105, USA. POWER_SAGE: Comparing statistical tests for SAGE experiments. *Bioinformatics* **16:** (11) 953.

Mayor C, Brudno M, Schwartz JR, Poliakov A, Rubin EM, Frazer KA, Pachter LS*, Dubchak I. 2000. *Univ Calif, Dept Math, Berkeley, Ca 94720, USA. *VISTA*: Visualizing global DNA sequence alignments of arbitrary length. *Bioinformatics* **16:** (11) 1046.

McClintick J, Edenberg HJ*. 2000. *Indiana Univ, Dept Biochem & Mol Biol, Indianapolis, In 46202, USA. BlastReport: A perl script to facilitate the use of sequence databases for mapping and clustering. *Biotechniques* **29:** (6) 1272.

Mizuguchi K, Blundell TL. 2000. Univ Cambridge, Dept Biochem, 80 Tennis Court Rd, Old Addenbrookes Site, Cambridge CB2 1QW, England. Analysis of conservation and substitutions of secondary structure elements within protein superfamilies. *Bioinformatics* **16:** (12) 1111.

Moler EJ, Chow ML, Mian IS*. 2000. *Univ Calif, Lawrence Berkeley Natl Lab, Dept Cell & Mol Biol, Berkeley, Ca 94720, USA. Analysis of molecular profile data using generative and discriminative methods. *Physiol Genomics* **4:** (2) 109.

Morgenstern B. 2000. Max-Planck-Inst Biochem, Klopferspitz 18A, DE-82152 Martinsried, Germany. A space-efficient algorithm for aligning large genomic sequences. *Bioinformatics* **16:** (10) 948.

Muilu J, Rodriguez-Tome P, Robinson A. 2001. European Bioinformat Inst, EMBL Outstn, Wellcome Trust Genome Campus, Hinxton, Cambridge CB10 1SD, England. GBuilder: An application for the visualization and integration of EST cluster data. *Genome Res* **11:** (1) 179.

Murvai J, Vlahovicek K, Pongor S. 2000. Int Ctr Genet Engn & Biotechnol, Prot Struct & Funct Grp, Area Sci Pk, Padriciano 99, IT-34012 Trieste, Italy. A simple probabilistic scoring method for protein domain identification. *Bioinformatics* **16:** (12) 1155.

Nishikawa T, Ota T, Isogai T. 2000. Hitachi Ltd, Biosyst Res Dept, 1-280 Higashi, Koigakubo, Kokubunji, Tokyo 185 8601, Japan. Prediction whether a human cDNA sequence contains initiation codon by combining statistical information and similarity with protein sequences. *Bioinformatics* **16:** (11) 960.

Page RDM. 2000. Univ Glasgow, Div Environm & Evolutionary Biol, Graham Kerr Bldg, Glasgow G12 8QQ, Scotland. Circles: Automating the comparative analysis of RNA secondary structure. *Bioinformatics* **16:** (11) 1042.

Park J, Dietmann S, Heger A, Holm L. 2000. European Bioinformat Inst, EMBL Outstn, Genome Campus, Cambridge CB10 1SD, England. Estimating the significance of sequence order in protein secondary structure and prediction. *Bioinformatics* **16:** (11) 978.

Pertsemlidis A, Pande A, Miller B, Schilling P, Wei MH, Lerman MI, Minna JD, Garner HR*. 2000. *UT SW Med Ctr, Dept Biochem, 5323 Harry Hines Blvd, Dallas, Tx 75390, USA. PANORAMA: An integrated web-based sequence analysis tool and its role in gene discovery. *Genomics* **70:** (3) 300.

Promponas VJ, Enright AJ, Tsoka S, Kreil DP, Leroy C, Hamodrakas S, Sander C, Ouzounis CA*. 2000. *European Bioinformat Inst, Res Programme, EMBL Cambridge Outstn, Cambridge CB10 1SD, England. CAST: An alternative algorithm for the complexity analysis of sequence tracts. *Bioinformatics* **16:** (10) 915.

Ramu C, Gemund C, Gibson TJ. 2000. EMBL, Meyerhofstr 1, Postfach 10-2209, Heidelberg, Germany. Object-oriented parsing of biological databases with Python. *Bioinformatics* **16:** (7) 628.

Rhee SY. 2000. Carnegie Inst Washington, Dept Plant Biol, 260 Panama St, Stanford, Ca 94305, USA. Bioinformatic resources, challenges, and opportunities using *Arabidopsis* as a model organism in a post-genomic era. *Plant Physiol* **124:** (4) 1460.

Rivas E, Eddy SR*. 2000. *Washington Univ, Dept Genet, St Louis, Mo 63110, USA. Secondary structure alone is generally not statistically significant for the detection of noncoding RNAs. *Bioinformatics* **16:** (7) 583.

Rutherford K, Parkhill J, Crook J, Horsnell T, Rice P, Rajandream MA, Barrell B. 2000. Wellcome Trust Genome Campus, Sanger Ctr, Hinxton, Cambridge CB10 1SA, England. Artemis: sequence visualization and annotation. *Bioinformatics* **16:** (10) 944.

Rzhetsky A, Koike T, Kalachikov S, Gomez SM, Krauthammer M, Kaplan SH, Kra P, Russo JJ, Friedman C. 2000. Columbia Univ, Genome Ctr, New York, NY 10027, USA. A knowledge model for analysis and simulation of regulatory networks. *Bioinformatics* **16:** (12) 1120.

Sakharkar MK, Kangueane P, Woon TW, Tan TW, Kolatkar PR, Long MY, De Souza SJ. 2000. Natl Univ Singapore, Bioinformat Ctr, SG-119260 Singapore, Rep Singapore. IE-Kb: Intron exon knowledge base. *Bioinformatics* **16:** (12) 1151.

Salamon H, Kato-Maeda K, Small PM, Drenkow J, Gingeras TR. 2000. Berlex Biosci, Dept Immunol, an Pablo Ave, Richmond, Ca 94804, USA. Detection of deleted genomic DNA using a semiautomated computational analysis of GeneChip data. *Genome Res* **10:** (12) 2044.

Sanchez-Pulido L, Yuan YP, Andrade MA, Bork P. 2000. CSIC, Ctr Nacl Biotechnol, Prot Design Grp, Campus Univ Autonoma, ES-28049 Madrid, Spain. NAIL: Network Analysis Interface for Linking HMMER results. *Bioinformatics* **16:** (7) 656.

Serra-Hartmann X, Rebordosa X, Pinol J, Querol E, Marti-Renom MA*. 2000. *Univ Autonoma, Inst Biol Fonamental, ES-08193 Bellaterra, Barcelona, Spain. ASAP: Analysis of peptide composition. *Bioinformatics* **16:** (12) 1153.

Silverstein KAT, Kilian A, Freeman JL, Johnson JE, Awad IA, Retzel EF. 2000. Univ Minnesota, Computat Ctr, 420 Delaware St SE, Minneapolis, Mn 55455, USA. PANAL: An integrated resource for Protein sequence ANALysis. *Bioinformatics* **16:** (12) 1157.

Vasconcelos AT, Maia MAGM, De Almeida DF. 2000. MCT, Lab Nacl Comp Cient, Rua Getulio Vargas 333, BR-25651-070 Petropolis, RJ, Brazil. Short interrupted palindromes on the extragenic DNA of *Escherichia coli* K12, *Haemophilus influenza* and *Neisseria meningitidis*. *Bioinformatics* **16:** (11) 968.

Vlahovicek K, Pongor S. 2000. Int Ctr Genet Engn & Biotechnol, Prot Struct & Funct Grp, Area Sci Pk, IT-34012 Trieste, Italy. *model.it*: Building three dimensional DNA models from sequence data. *Bioinformatics* **16:** (11) 1044.

Voit EO, Radivoyevitch T. 2000. Med Univ Sth Carolina, Dept Biometry & Epidemiol, 135 Rutledge Ave, Charleston, SC 29425, USA. Biochemical systems analysis of genome-wide expression data. *Bioinformatics* **16:** (11) 1023.

Wallqvist A, Fukunishi Y, Murphy LR, Fadel A, Levy RM. 2000. Rutgers State Univ, Dept Chem, 610 Taylor Rd, Piscataway, NJ 08854, USA. Iterative sequence/secondary structure search for protein homologs: Comparison with amino acid sequence alignments and application to fold recognition in genome databases. *Bioinformatics* **16:** (11) 988.

Wheelan SJ, Marchler-Bauer A, Bryant SH*. 2000. NIH/Natl Lib Med, Natl Ctr Biotechnol Informat, Bethesda, Md 20894, USA. Domain size distribution can predict domain boundaries. *Bioinformatics* **16:** (7) 613.

Wheeler R, Hughey R*. 2000. *Univ Calif, Jack Baskin Sch Engn, Dept Comp Engn, Santa Cruz, Ca 95064, USA. Optimizing reduced-space sequence analysis. *Bioinformatics* **16:** (12) 1082.

Woodwark KC, Hubbard SJ, Oliver SG. 2001. UMIST, Sch Biol Sci, Oxford Rd, Manchester M60 1QD, England. Sequence search algorithms for single pass sequence identification: Does one size fit all? *Comp Funct Genom* **2:** (1) 4.

Xu Y, Xu D, Gambow HN. 2000. Oak Ridge Natl Lab, Div Life Sci, Computat Biosci Sect, Oak Ridge, Tn 37830, USA. Protein domain decomposition using a graph-theoretic approach. *Bioinformatics* **16:** (12) 1091.

Ye SQ, Zhang LQ, Zheng F, Virgil D, Kwiterovich PO. 2000. Johns Hopkins Univ, Dept Pediat, 600 Nth Wolfe St, Baltimore, Md 21287, USA. miniSAGE: Gene expression profiling using serial analysis of gene expression from 1 μg total RNA. *Anal Biochem* **287:** (1) 144.

Zhu H, Schein CH, Braun W*. 2000. *UT Med Branch, Sealy Ctr Struct Biol, Galveston, Tx 77555, USA. MASIA: Recognition of common patterns and properties in multiple aligned protein sequences. *Bioinformatics* **16:** (10) 950.

Copyright © 2001 John Wiley & Sons, Ltd. 
